# A Protein L -Based Immunodiagnostic Approach Utilizing Time-Resolved Förster Resonance Energy Transfer

**DOI:** 10.1371/journal.pone.0106432

**Published:** 2014-09-02

**Authors:** Satu Hepojoki, Visa Nurmi, Antti Vaheri, Klaus Hedman, Olli Vapalahti, Jussi Hepojoki

**Affiliations:** 1 Department of Virology, Haartman Institute, University of Helsinki, Helsinki, Finland; 2 Helsinki University Central Hospital, Hospital District of Helsinki and Uusimaa, Laboratory Services, HUSLAB, Helsinki, Finland; 3 Department of Veterinary Biosciences, University of Helsinki, Helsinki, Finland; CNR, Italy

## Abstract

Chelated lanthanides such as europium (Eu) have uniquely long fluorescence emission half-lives permitting their use in time-resolved fluorescence (TRF) assays. In Förster resonance energy transfer (FRET) a donor fluorophore transfers its emission energy to an acceptor fluorophore if in sufficiently close proximity. The use of time-resolved (TR) FRET minimizes the autofluorescence of molecules present in biological samples. In this report, we describe a homogenous immunoassay prototype utilizing TR-FRET for detection of antibodies in solution. The assay is based on labeled protein L, a bacterial protein that binds to immunoglobulin (Ig) light chain, and labeled antigen, which upon association with the same Ig molecule produce a TR-FRET active complex. We show that the approach is functional and can be utilized for both mono- and polyvalent antigens. We also compare the assay performance to that of another homogenous TR-FRET immunoassay reported earlier. This novel assay may have wide utility in infectious disease point-of-care diagnostics.

## Introduction

Biological sample materials are prone to autofluorescence, which can be minimized by utilizing time-resolved fluorometry (TRF). TRF takes advantage of unique rare earth elements called lanthanides, such as europium, which have exceptionally long fluorescence emission half-lives. In Förster resonance energy transfer (FRET), energy is transferred between two fluorophores, the donor and the acceptor [1]. Time-resolved FRET (TR-FRET) unites the properties of TRF and FRET, which is especially advantageous when analyzing biological samples. As TR-FRET -based methods induce relatively low background fluorescence, this technique has been widely applied in medical research and diagnostics [2]–[10]. Overall, TR-FRET -based applications offer a viable alternative for the conventional multistep diagnostic tests, such as enzyme-linked immunosorbent assay (ELISA).

We recently developed for detection of antibodies a TR-FRET -based homogeneous immunoassay denoted “FRET-bridge” which employs antigens separately labeled with donor- and acceptor-fluorophores [11]. In the FRET-bridge assay simultaneous binding of donor- and acceptor-labeled antigens to an immunoglobulin (Ig) G molecule can be measured by TR-FRET. The FRET-bridge assay was set up utilizing a tetrameric antigen, streptavidin (SA), due to its commercial availability with fluorescent labels. However, when evaluating the performance of FRET-bridge assay using monovalent antigens, we observed decreased sensitivity. The FRET-bridge assay requires each antigen to be separately labeled with two fluorophores, which is expensive and also potentially hampered by the random attachment of the labels that might affect the immunologically important epitopes. As the efficiency of FRET is dependent on the distance between the donor and acceptor fluorophores, we reasoned that in an ideal TR-FRET assay both of the two fluorophores should preferentially bind to the same Fab-arm of the Ig molecule. This could not only improve the assay sensitivity by bringing the interacting fluorophores closer, but would also reduce the number of labeling reactions required for multiple assays, because the same Ig-binding molecule could be used in combination with a variety of antigens. In addition, one IgG molecule could form two FRET-pairs one with each Fab-arm, thus likely increasing the signal intensity.

To implement the above-mentioned idea, we chose to use protein L, a bacterial surface protein originally derived from *Finegoldia (formerly Peptostreptococcus) magnus* [12] as the Ig-binding molecule. Protein L binds the Ig kappa (κ) light chain without interfering with the antigen recognition [12]. Through κ light chain interaction protein L is capable of binding to all immunoglobulin classes (IgG, IgM, IgA, IgE and IgD) [13]. Additionally, protein L binds to single-chain variable fragments (scFv) and Fab fragments bearing κ light chains [14], [15]. Therefore, protein L binds to a wider range of Ig classes and subclasses than the other antibody-binding proteins, such as protein A and G [16], [17].

We chose to use europium-chelate (Eu) as the donor due to its spectral properties. Fluorescent Eu-chelates exhibit large Stoke's shifts with no overlap between the excitation (at 320 nm in our assay) and emission (at around 615 nm) wavelenghts. The emission wavelength is above the background fluorescense from biological samples (usually 300–600 nm) [18]. For FRET to occur, spectral overlap of the donor emission and acceptor absorption is required. AlexaFluor647 (AF647, excitation maximum at 650 nm) is commonly used as a FRET-pair for Eu, due to spectral overlap of these fluorophores.

Here, we describe a novel approach for detection of antibodies in solution. The approach relies on fluorophore-labeled recombinant protein L, which in conjunction with fluorophore-labeled antigen induce TR-FRET signal in the presence of antibody specific to the antigen. This novel approach is simpler than the FRET-bridge assay [11], because it requires the antigen to be labeled with only a single fluorophore. Moreover, the assay based on labeled protein L is highly versatile, detecting all antibody classes in combination with a practically unlimited range of antigens. Herein we provide the proof-of-principle and determine the assay performance for the novel approach utilizing both monomeric and tetrameric antigens. In addition, we compare the performances of the protein L and FRET-bridge immunoassays with particular emphasis on the mono- versus multimeric property of the antigen.

## Materials and Methods

### Ethics statement

The human serum sample used in this study was obtained under the research permit of ethical committee of Hospital District of Helsinki and Uusimaa, number 553/E6/2001. A written informed consent was obtained from the donor.

### Proteins and antibodies

Recombinant protein L was purchased from Thermo Scientific (Pierce Protein Biology Products) and separately labeled with QuickAIIAssay Eu-chelate protein labeling kit (BN products & Services Oy) and Alexa Fluor 647 (AF647) Protein Labeling Kit (Invitrogen). The labeling was performed according to the manufacturers' instructions. AF647 and Eu-chelate -labeled streptavidins (AF647-SA and Eu-SA, respectively) were ordered from Invitrogen and PerkinElmer, respectively. The unique region of human parvovirus B19 (B19V) minor capsid protein VP1 as a GST-fusion protein (here denoted as GST-VP1u) was expressed in E. coli and purified for serodiagnostic use [19], and independently labeled with Eu (using the QuickAIIAssay Eu-chelate protein labeling kit of BN Products & Services Oy) and AF647 (using the Protein Labeling Kit of Invitrogen), using the same protein stock, according to the manufacturers' instructions. GST-VP1u served as antigen for experiments with anti-GST antibodies because its molecular weight (53 kDa) is close to that of tetrameric SA. Monoclonal antibody (MAb) against SA (anti-SA) (clone S3E11, 6.1 mg mL-1) was purchased from Thermo Scientific (Pierce Protein Biology Products). Monoclonal anti-GST was from Abcam Ltd (1 mg mL-1) and IgG-free bovine serum albumin (BSA) from Jackson ImmunoResearch Inc. SA used for ELISA-coating was from New England Biolabs. Human IgG fraction was purified from human serum by High Trap protein G column (GE Healthcare) according to the instructions. Secondary antibodies, anti-mouse-HRP and anti-rabbit-HRP, were from Dakocytomation (P0260 and P0217, respectively). The human IgG-fraction was purified from serum using GammaBind Plus Sepharose (GE Healthcare) according to the manufacturers' instructions and under a research permit (553/E6/2001).

### Enzyme-linked immunosorbent assay (ELISA)

ELISA was done as described [11] on microwell strips (96-well format) coated overnight by 100 µl of GST-VP1u (1.5 µg mL−1) or 100 µl of SA (1.5 µg mL−1) in Tris-buffered saline (TBS, 50 mM Tris-HCl, 150 mM NaCl, pH 7.4). Wells were blocked with TBS containing 0.2% BSA (TBS-BSA). Three-fold dilution series (from 20 nM to 0.03 nM) of the monoclonal and polyclonal anti-GST and anti-SA antibodies were done in TBS-BSA.

### TR-FRET assays

The basic protocol for all protein L TR-FRET assays was as follows: after the reaction components were diluted in TBS-BSA, protein L was mixed with MAb, incubated for 15 min at +37°C and mixed with labeled SA. The final reaction volume (20 µl/well) was pipetted onto a 384-well microplate (ProxiPlate-384 Plus F, Black 384-shallow well Microplate from PerkinElmer). All experiments were performed in duplicate, and the TR-FRET values were measured with Wallac Victor^2^ fluorometer (PerkinElmer) using the measurement and normalization protocol described [11]. Also, protocol for the “FRET-bridge” assays was as described [11].

### Fab-fragment assays

Fab-fragments were generated from anti-SA MAb and purified with a described protocol [11]. The TR-FRET signals induced by the Fab-fragments in two-fold dilutions (200 nM to 50 nM) were compared to those induced by intact anti-SA MAb (50 nM to 12.5 nM). Both dilution series were mixed in equal volumes with solution containing 20 nM AF-labeled protein L and 20 nM Eu-SA. We used 20 nM AF-labeled protein L and 20 nM Eu-SA as control (no antibody).

## Results and Discussion

### Detection limits of the antibodies in ELISA

**Figure 1:**
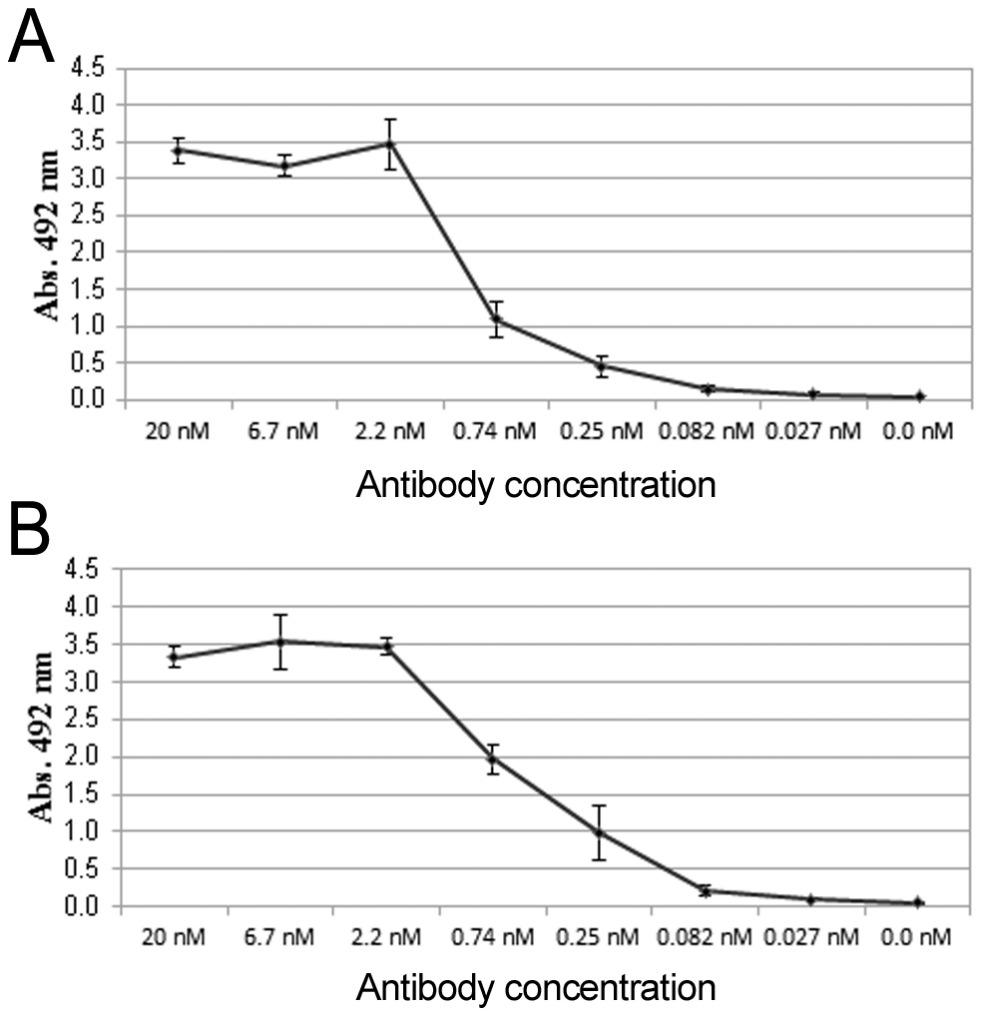
Antibody titration in ELISA. Monoclonal **A**) SA and **B**) GST antibodies were titrated in SA- or GST coated ELISA. The error bars represent ± standard deviation between parallel wells.

To determine whether multimericity of antigens affects the performance of TR-FRET assays, we used GST-VP1u and SA (tetrameric) as antigens. We first compared the detection limits of the anti-GST and anti-SA MAbs in ELISA. The lowest concentrations that produced a signal above background were 0.08 nM for both of the two MAbs (Fig. 1A and B, respectively). These results imply that the sensitivities of both MAbs are roughly equal.

### Proof of principle - Binding of protein L and antigen to the same antibody is detectable by TR-FRET

**Figure 2:**
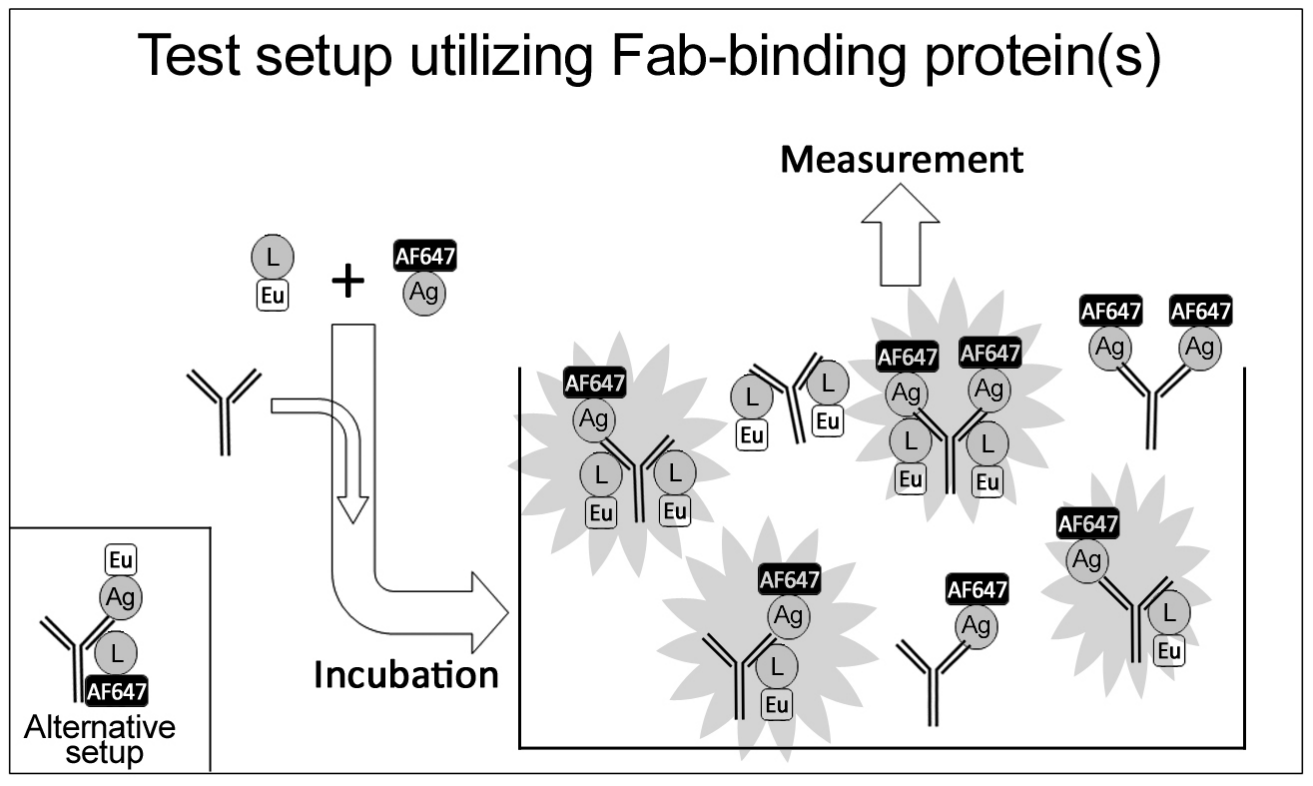
A schematic overview of the assay procedure.

We have shown that mixtures of SA labeled separately with Eu (donor) and AF647 (acceptor) combined with mono- or polyclonal anti-SA readily induce TR-FRET [11]. Since TR-FRET signal is proportional to the distance between donor and acceptor, we explored the possibility of using a generic Fab-binding molecule and a labeled antigen as constituents of a TR-FRET -based homogeneous immunoassay. The experimental setup is illustrated in Figure 2. After combining the reagents, the interacting molecules form complexes of various types and sizes. For example, one IgG can bind protein L without the antigen (and vice versa), while another IgG binds both molecules. A single IgG can even bind two antigen-protein-L pairs, one with each Fab-arm, resulting in the formation of two FRET-pairs. To test the above hypothesis, protein L was used as the Fab-binding moiety and SA and GST-VP1u as the antigens. Additionally, we wanted to know if positioning of the labels (donor in protein L and acceptor in antigen vs. acceptor in protein L and donor in antigen) would affect the assay performance. We performed a set of cross-titration experiments, in which the amount of protein L was varied (100 nM–6.3 nM) against constant concentrations of the antibodies and antigens (10 nM and 20 nM, respectively). The experiment was performed in four different setups: AF-L + Eu-GST-VP1u, AF-L + Eu-SA, Eu-L + AF-GST-VP1u and Eu-L + AF-SA. In each setup the protein L + antigen combination was tested with a specific and a control antibody. In the experiments with anti-SA MAb, anti-GST MAb served as control, and vice versa. Also a buffer control was used, which included labeled protein L and antigen, but no antibody.

**Figure 3:**
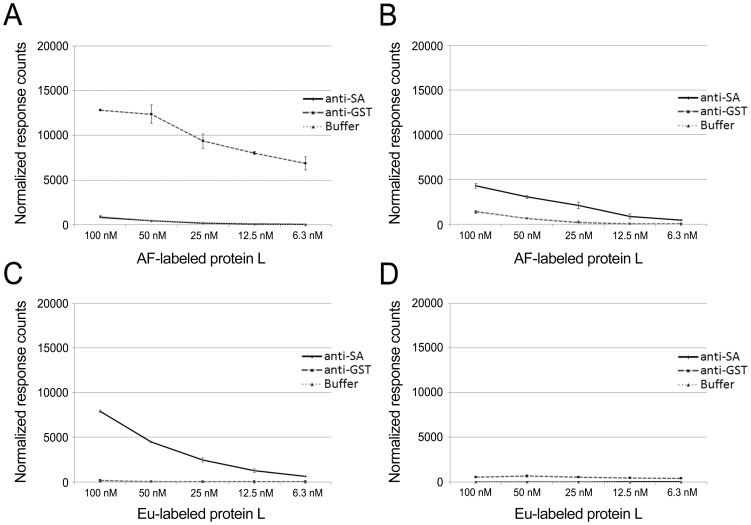
Eu- and AF-labeled protein L titration assays. AF-labeled protein L was titrated (100–6.3 nM) separately with **A**) Eu-labeled GST-VP1u and **B**) Eu-labeled SA. Eu-labeled protein L was titrated (100–6.3 nM) separately with **C**) AF-labeled SA and **D**) AF-labeled GST-VP1u. The anti-GST antibody was used as control for SA and anti-SA antibody for GST-VP1u. The third line represents a background control without antibody. The y-axis represents response counts obtained from Victor fluorometer. The error bars represent ± standard deviation between parallel wells.

With AF-labeled protein L and Eu-labeled antigens (GST-VP1u and SA), both of the antigens induced TR-FRET signals dose-dependently on protein L concentration; however, the signals induced by the Eu-GST-VP1u (Fig. 3A) were significantly higher (>10-fold over background) than those induced by the Eu-SA (Fig 3B) (∼3-fold over background). The control antibodies (anti-SA and anti-GST) induced background comparable to that without antibody (Fig. 3A and 3B). Both background signals were proportional to the AF-label concentration.

With Eu-labeled protein L in combination with AF-labeled antigens, only AF-SA (Fig. 3C) induced an unequivocally dose-dependent increase in signal, while AF-GST-VP1u (Fig. 3D) induced a low TR-FRET-response. Very low background fluorescence, proportional to Eu concentration, was observed with the control antibodies and in the absence of antibodies (Fig. 3C and 3D).

The above results provide a proof-of-principle for the approach depicted in Figure 2. Furthermore, the results indicate that increasing the concentration of protein L in the reaction mixture in proportion to IgG produces higher TR-FRET signals, but also increases background thus resulting in lower signal to noise (S/N) ratios. However, even at the highest concentrations there was an unequivocal signal-to-noise difference. Remarkably, the assay performance was better (with both GST-VP1u and SA) when protein L was labeled with AF (acceptor) than with Eu (donor). Increasing the amount of acceptor-labeled molecule combined with the time-resolved measurement does not affect the S/N ratio to the same extent as increasing the amount of donor-labeled molecule, which could partly explain the results. Also, changes in the binding affinity (induced by labeling) of either protein L or the antigens, and the degree of labeling (DOL) (number of fluorophores, Eu or AF, per molecule) might affect the TR-FRET signal intensities. The DOL was between 3 and 4 for all molecules except for Eu-SA (DOL of 6-7). Since the molecules differ in their sizes, the optimization of DOL might improve the results. Also the positioning of the label in the molecule might affect the results by increasing or decreasing the distance between the interacting fluorophores. Both GST-VP1u and protein L contain >40 lysine residues potentially reactive with the label. Unfortunately, the labeling chemistries for Eu and SA are different, whereby the two may be incorporated in different lysine residues. This hampers exact control over the FRET-distance between these fluorophores. It is encouraging to see that a rather large excess (as high as 100 nM) of protein L can be used in the assay, for it to be functional in human serodiagnostics. As the proportions of specific IgG in acute-phase sera are typically low (<1-5%) and variable, an excess of protein L might be required to “saturate” the IgG molecules (of all specificities) in the sample.

### Optimization of the protocol by cross-titration

**Figure 4:**
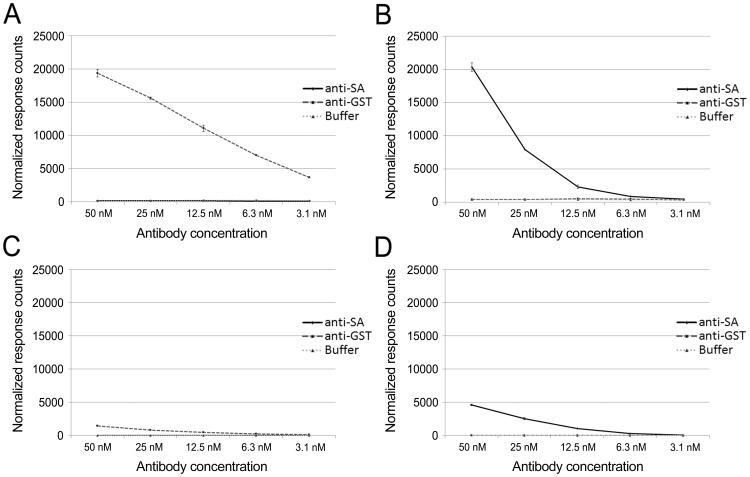
Antibody titration assays using AF-labeled or Eu-labeled protein L. **A**) Anti-GST antibody titrated against Eu-labeled GST-VP1u and AF-labeled protein L. **B**) Anti-SA antibody titrated against Eu-labeled SA and AF-labeled protein L. **C**) Anti-SA antibody titrated against AF-labeled SA and Eu-labeled protein L. **D**) Anti-GST antibody titrated against AF-labeled GST-VP1u and Eu-labeled protein L. In all setups antibody concentrations were from 3.1 nM to 50 nM, and the antigen and protein concentration was constant (20 nM). Anti-GST antibody was used as a control for SA assays, and anti-SA for GST assays. The third line represents a background control with no antibody. The y-axis represents response counts obtained from Victor^2^ fluorometer. The error bars represent ± standard deviation between parallel wells.

To assess the optimal amounts of protein L and antigens relative to antibody concentrations, we titrated the antibodies (anti-GST and anti-SA at 50 nM–3.1 nM) against constant concentrations of the antigens (Eu- or AF-labeled SA and GST-VP1u at 20 nM) and protein L (Eu- or AF-labeled at 20 nM). With AF-labeled protein L and Eu-labeled antigens both of the two antigens induced TR-FRET signals (Fig. 4A and 4B) dose-dependently with antibody concentrations. However, when the donor label (Eu) was in protein L, AF-SA (Fig. 4C) induced markedly higher TR-FRET responses than did AF-GST-VP1u (Fig. 4D), as noted above. No TR-FRET signals were detected in the presence (or absence) of the control antibodies in either experimental setup (AF-L and Eu-L, Fig. 4A–D).

The signal to background ratio (18-fold) at the end point (3.1 nM, Fig. 4A) of anti-GST antibody titration indicates that the protein L assay could detect even lower antibody concentrations than what was tested. Because the sensitivities with the other antigen-antibody combinations were not as good, we decided not to continue titration to lower antibody concentrations. The ELISA end-point titration of anti-GST antibody showed detection sensitivity approximately 40 times higher than with our assay (0.08 nM vs. 3.1 nM). However, the total assay time with ELISA (roughly 4 h) is considerably longer than that with protein L TR-FRET assay (<30 min). The level of IgG in human serum is ∼5–15 mg/ml [20], of which antibody of a particular specificity may comprise 0.5–2 µM, several 100-fold over the detection limit of our assay.

**Figure 5:**
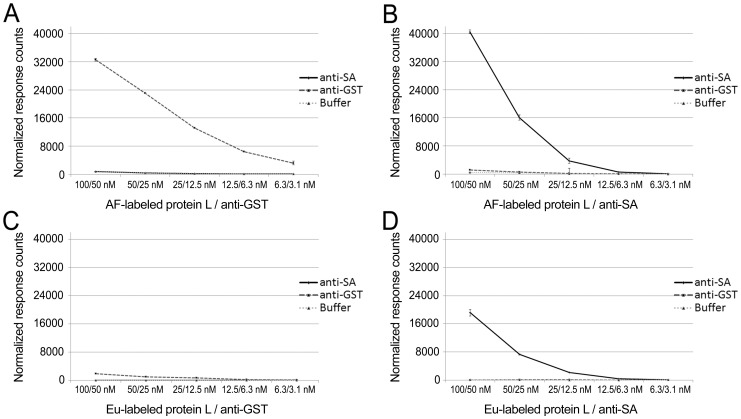
Titration of protein L and antibodies against constant antigen concentration. **A**) AF-labeled protein L and anti-GST antibody titrated against Eu-labeled GST-VP1u. **B**) anti-SA and AF-labeled protein L titrated against Eu-labeled SA. **C**) Anti-GST antibody titrated together with Eu-labeled protein L against AF-labeled GST-VP1u. **D**) Anti-SA antibody titrated together with Eu-labeled protein L against AF-labeled SA. In all experiments the antigen concentration was kept at 20 nM, and the amount of protein L was two times more than the amount of antibody. Anti-GST antibody was used as a control for SA assays, and anti-SA for GST assays. The third line represents a background control with no antibody. The y-axis represents response counts obtained from Victor^2^ fluorometer. The error bars represent ± standard deviation between parallel wells.

To complete the cross-titration experiments, we serially diluted protein L and antibodies together (100 nM/50 nM–6.3 nM/3.1 nM, respectively) against constant concentrations of the antigens (20 nM). A dose-dependent signal increase was observed when increasing the concentrations of the antibodies and protein L. And again, the experiments with AF-L in combination with Eu-labeled antigens (Fig. 5A and B) gave higher TR-FRET signals compared to Eu-labeled protein L in combination with AF-labeled antigens (Fig. 5C and D). No TR-FRET signals were observed in the presence or absence of control antibodies (Fig. 5A–D).

Eu-labeling is more expensive and demanding than AF-labeling, whereby it could be desirable to have the Eu-label in the protein L. Then, the same batch of protein L could be used in combination with different microbial antigens labeled with AF. However, according to our cross-titration experiments with the two antigens, SA and GST-VP1u, it seems beneficial for the assay performance to have protein L carry the acceptor. This is mostly because increasing the acceptor concentration in the reaction mix does not increase the background as heavily as increasing the donor concentration does.

### Assay performance in the presence of unrelated IgG

**Figure 6:**
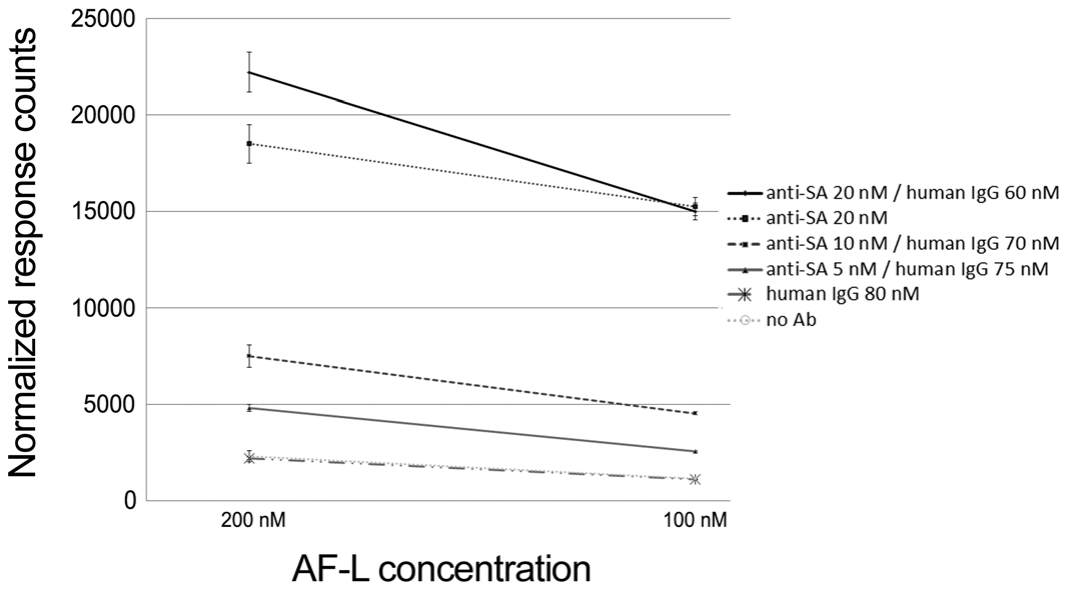
TR-FRET signals induced by anti-SA antibody diluted in human IgG at various concentrations. Anti-SA Antibody was diluted in human IgG 1/4, 1/8 and 1/16, and tested with two dilutions of AF-labeled protein L: 100 nM and 200 nM, while the antigen concentration was kept constant (10 nM). Dilutions of anti-human IgG (80 nM), anti-SA (20 nM) were used as antibody controls, and buffer with AF-L and Eu-SA at 10 nM was used as background control. The y-axis represents response counts obtained from Victor^2^ fluorometer. The error bars represent ± standard deviation between parallel wells.

The assay described herein relies on binding of both protein L and antigen to a single IgG molecule. As discussed, only a fraction of the IgG molecules in serum is specific for a given antigen while the remaining specificities “consume” the protein L. We evaluated the assay performance with regard to the presence of non-related human IgG, by mixing anti-SA antibody with an IgG fraction of non-immune human serum in three different ratios: 1/4 (20 nM anti-SA, 80 nM human IgG), 1/8 (10 nM anti-SA, 70 nM human IgG) and 1/16 (5 nM anti-SA, 75 nM human IgG). Anti-SA antibody (at 20 nM) alone and human IgG (at 80 nM) alone served as controls. The antigen (Eu-SA) concentration was kept constant (10 nM), while the protein L concentration was varied (100 nM to 200 nM). As shown in Figure 6, anti-SA antibody mixed with non-related IgG at 1/4 ratio gave essentially the same TR-FRET signal intensity as anti-SA antibody alone. The signals obtained at 1/8 ratio were approximately 30% lower than those at 1/4 ratio, whereas those at 1/16 were approximately 60% lower than those at 1/4. Altogether, the results indicate that even low amounts of antibody (1/16 dilution, corresponding to 5 nM specific IgG) can be detected using the new assay, using ample amounts of AF-labeled protein L.

### Protein L and antigen mixed with Fab-fragment produce TR-FRET signal

**Figure 7:**
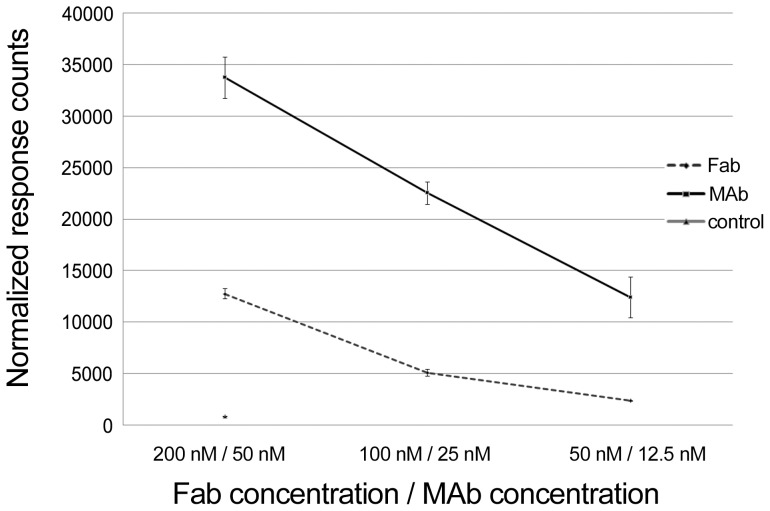
The performance of Fab-fragments generated from anti-SA MAb in the protein L assay. Fab-fragments were diluted from 200 nM to 50 nM, and a dilution series (50 nM to 12.5 nM) of anti-SA MAb acting as control was tested in parallel. The third line represents a control for fluorescence background induced by AF-labeled protein L and Eu-SA. The y-axis represents response counts obtained from Victor^2^ fluorometer. The error bars represent ± standard deviation between parallel wells.

We next determined whether TR-FRET signals induced by labeled protein L and antigen can originate from the same Fab-arm of the IgG molecule. To demonstrate that steric hindrance does not prevent simultaneous binding of protein L and antigen, we prepared anti-SA Fab-fragments by papain cleavage, and performed titration series. The intact anti-SA MAb was used as positive control. We compared the signals induced by Fab-fragments to signals induced by intact antibodies. Since we have generated Fab-fragments from an anti-SA MAb [11], we used SA as antigen in this setup. We serially diluted the anti-SA Fab-fragments and antibodies and mixed them separately with labeled protein L and SA. As shown in Figure 7, the Fab fragments produced a dose-dependent increase in TR-FRET signals, analogously to the signals induced by the MAb. The signal intensities obtained with Fab-fragments were lower than those obtained with the intact MAb, which might be due to structural damage due to papain. Alternatively, the divalent of nature of intact MAb could improve signal intensities due to the diversity of FRET-active complexes formed (see Fig. 2). Altogether, this experiment demonstrates that protein L and antigen can form a FRET-pair within a single Fab.

### Protein L -based vs. “FRET-bridge” immunoassay

Finally, to compare the protein L mediated assay to the FRET-bridge approach based on donor-labeled and acceptor-labeled antigens [11], we compared the two at identical antibody and antigen concentrations. Additionally, we examined the performances of both assays using mono- and tetravalent antigens (GST-VP1u and SA, respectively). The concentrations of the corresponding MAbs (anti-SA and anti-GST) and of the AF-labeled protein L were varied, while that of the antigens (Eu-SA and Eu-GST-VP1u) was kept constant (10 nM). The antibody concentrations ranged from 3.1 nM to 25 nM, while the protein L concentration was always two-fold higher than that of the antibody (6.3 nM to 50 nM). We used AF-labeled protein L (6.3 nM to 50 nM) and 20 nM SA as control (no antibody).

**Figure 8:**
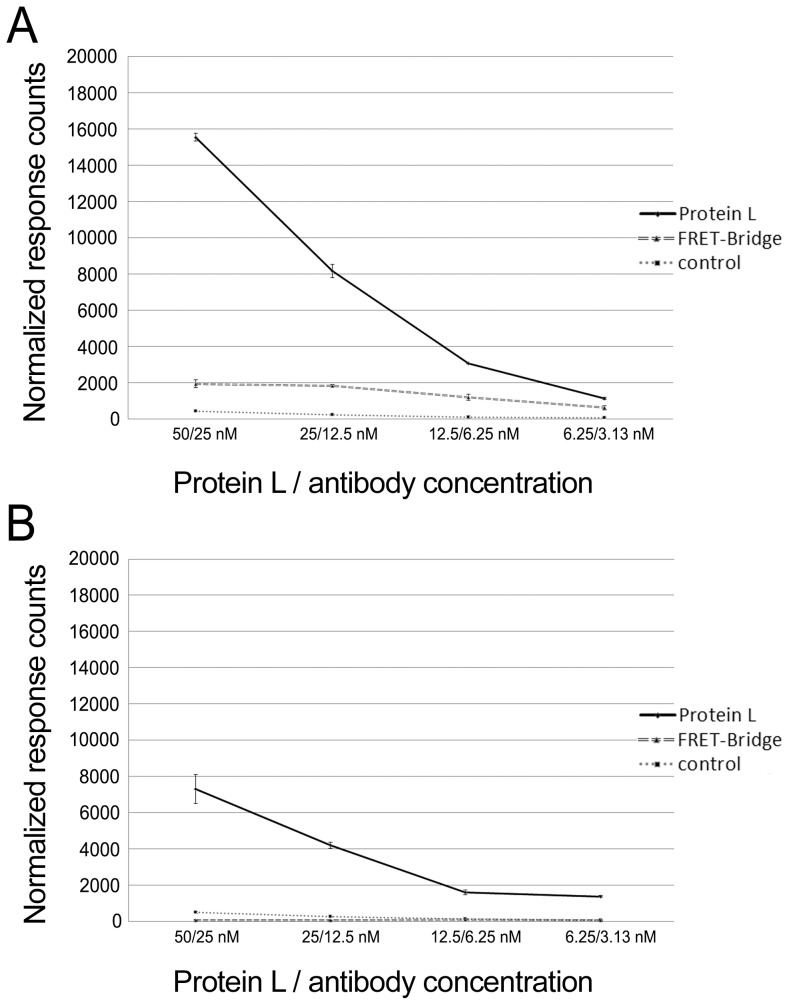
Protein L vs. FRET-bridge assay. Dilutions series (25–3.1 nM) of the antibodies, A) anti-SA antibody and B) anti-GST, were tested in parallel with both of the assay approaches. Protein L was titrated along with the antibody concentration, representing concentrations from 50 to 6.3 nM. Concentrations of GST-VP1u and SA were constant at 10 nM. The third line in both figures represents a control for fluorescence background induced by AF-labeled protein L and the antigen. The y-axis represents response counts obtained from Victor^2^ fluorometer. The error bars represent ± standard deviation between parallel wells.

Both of the antigens, SA and GST-VP1u, induced higher TR-FRET signals in the protein L assay (Fig. 8A and B). In fact, the GST-VP1u antigen did not induce any signal in the FRET-bridge assay, in line with our previous notion [11] that in the FRET-bridge assay the multimericity of the antigen is beneficial. We have observed that most of the TR-FRET activity in the FRET-bridge assay is derived from large immunocomplexes consisting of more than one IgG and two antigens [11]. We assume that the formation of these highly TR-FRET-active immunocomplexes is, to some extent, hindered by monomeric antigens. In theory, a tetrameric antigen should bind more antibodies than a monomeric antigen, at least in case of MAb. In the protein L assay, SA induced twice higher TR-FRET signals compared to GST-VP1u. Hence, it seems that also in the protein L approach, multimericity of the antigen is beneficial, although the higher DOL of Eu-SA might also explain the observed difference in performance of the two antigens. One reason for the induction of higher TR-FRET responses in the protein L assay could be that the recombinant protein L contains four κ light chain-binding domains. However, it is possible that binding of several Igs by a single protein L molecule would be sterically hindered.

## Conclusions

Herein we describe a novel TR-FRET -based immunoassay that relies on simultaneous binding of fluorophore-labeled protein L and fluorophore-labeled antigen to an IgG molecule. We show that the assay principle is functional, and that the FRET signal can be generated by binding of the protein L and antigen to the same Fab arm of IgG. We also demonstrate that the labeled protein L in excess can be used to “saturate” a mixture of IgG molecules, enabling the use of this approach in serodiagnostics. Comparison of the new assay to the previously reported FRET-bridge assay showed the former to be more sensitive and less dependent on antigen multimericity. The main advantages of protein L assay as compared to commonly utilized immunological assays such as ELISA, are the assay time (Protein L assay ∼30 minutes vs. ELISA ∼4 h), the wash-free format, and the ease of performance. After providing this proof-of-concept for the test system using readily accessible materials we will adapt the approach for serodiagnostics of infectious diseases.
